# Landscape-scale mapping of soil fungal distribution: proposing a new NGS-based approach

**DOI:** 10.1038/s41598-023-37538-7

**Published:** 2023-06-24

**Authors:** Daniel Janowski, Tomasz Leski

**Affiliations:** 1 Institute of Dendrology, Polish Academy of Sciences, Kórnik, Poland; 2 Department of Natural Environmental Studies, The University of Tokyo, Kashiwa, Chiba Japan

**Keywords:** Biodiversity, Biogeography, Community ecology, Conservation biology, Microbial ecology

## Abstract

Soil fungi play an indispensable role in the functioning of terrestrial habitats. Most landscape-scale studies of soil fungal diversity try to identify the fungal taxa present at a study site and define the relationships between their abundance and environmental factors. The specific spatial distribution of these fungi over the site, however, is not addressed. Our study’s main objective is to propose a novel approach to landscape-scale mapping of soil fungi distribution using next generation sequencing and geographic information system applications. Furthermore, to test the proposed approach and discuss its performance, we aimed to conduct a case study mapping the spatial distribution of soil fungi on the Wielka Żuława island. The case study was performed on the Wielka Żuława island in northern Poland, where soil samples were collected every 100 m in an even grid. The fungal taxa and their relative abundance in each sample were assessed using the Illumina platform. Using the data obtained for the sampled points, maps of soil fungi spatial distribution were generated using three common interpolators: inverted distance weighted (IDW), B-spline, and ordinary Kriging. The proposed approach succeeded in creating maps of fungal distribution on Wielka Żuława. The most abundant groups of soil fungi were *Penicillium* on the genus level, Aspergillaceae on the family level, and ectomycorrhizal fungi on the trophic group level. Ordinary Kriging proved to be the most accurate at predicting relative abundance values for the groups of fungi significantly spatially autocorrelated at the sampled scale. For the groups of fungi not displaying spatial autocorrelation at the sampled scale, IDW provided the most accurate predictions of their relative abundance. Although less accurate at predicting exact relative abundance values, B-spline performed best in delineating the spatial patterns of soil fungi distribution. The proposed approach to landscape-scale mapping of soil fungi distribution could provide new insights into the ecology of soil fungi and terrestrial ecosystems in general. Producing maps of predicted fungal distribution in landscape-scale soil fungi diversity studies would also facilitate the reusability and replicability of the results. Outside the area of research, mapping the distribution of soil fungi could prove helpful in areas such as agriculture and forestry, nature conservation, and urban planning.

## Introduction

Soil microorganisms are a crucial component of terrestrial ecosystems. They are fundamental in sustaining soil fertility, nutrient cycling, and plant growth^1,2^. Although bacteria dominate soil microbial communities^3,4^, the importance of fungi cannot be overstated^2,5^. One of the essential roles unique to soil fungi is forming mycorrhizal symbiosis with plants^6^. Several fungal saprotrophs specialize in decomposing recalcitrant organic compounds (e.g., lignin) that are not readily available to bacterial decomposers^7,8^. Fungi are also more effective than bacteria in carbon sequestration^9^. Despite the undeniable importance of microorganisms, they are relatively understudied, and aspects of their ecology remain unclear^10^.

Studying the distribution of soil fungi on different scales provides invaluable insight into soil ecology. In recent years we saw several studies of the global-scale distribution of soil fungi^4,11–13^. These studies helped reveal how large-scale effects (e.g., climate) shape fungal biogeography and increased our understanding of typical environmental preferences characterizing important fungal groups. While global-scale studies provide widely applicable insights, they do not reveal the fungal diversity and distribution patterns on regional^14,15^ and landscape^16,17^ scales. Understanding the landscape scale is particularly important for sustainable land use and conserving rare and endangered species^18^.

Rather than mapping fungi, landscape-scale studies of soil fungal diversity often concentrate on the observed correlations and trends^16,17,19^. In addition to the local soil fungal communities, selected environmental variables are measured to investigate whether they show significant effects on the recorded fungal distribution. If these observations have external validity, they advance the general understanding of soil fungal ecology. However, this approach reveals nothing about the actual distribution of soil fungi in the researched landscape. A continuous map of the local distribution of fungi would have several benefits. First, it might be useful for organizing future research at the same site. It would ease the replication crisis in ecology by enabling researchers to confirm each other’s findings more easily^20,21^. It might facilitate spotting any previously overlooked connections between the distribution of soil fungi and environmental factors. It could serve as a valuable tool in studying complex ecological networks involving soil fungi, e.g., mycorrhizal networks^19,22^. Finally, it would be invaluable for the conservation efforts of endangered fungi and some mycorrhizal plants.

Of course, it would not be practical to collect samples from each point of a studied landscape. However, mapping a continuous variable can be achieved through spatial interpolation. Interpolation involves using mathematical models to predict unknown variable values based on a finite number of known data points. Many spatial interpolation methods based on different mathematical models have been developed^23^. Commonly used methods include nearest- and natural-neighbor interpolations, inverse distance weighting (IDW), variations of Kriging interpolation, and variations of spline interpolation. It is not always clear which interpolation method is best for a given environmental variable; this depends on the patterns in that variable’s distribution and the sampling design, among others. Thus, comparing the effectiveness of interpolation methods is an important element of optimizing environmental variables mapping protocols^24–27^.

Our primary objective is to present a new method for local-scale distribution mapping of soil fungi. This approach combines Next Generation Sequencing (NGS) metabarcoding and geographic information system (GIS) applications involving spatial interpolation. The method can be used to map the distribution of one selected, studied group of fungi, as well as multiple groups or soil fungi in general. Our approach was tested in situ, and example fungal distribution maps were developed based on Wielka Żuława island, Poland’s largest inland island. Finally, we discuss the drawbacks of our approach and some possible adaptations and workarounds.

## Materials and methods

### Study site and sampling

**Figure 1:**
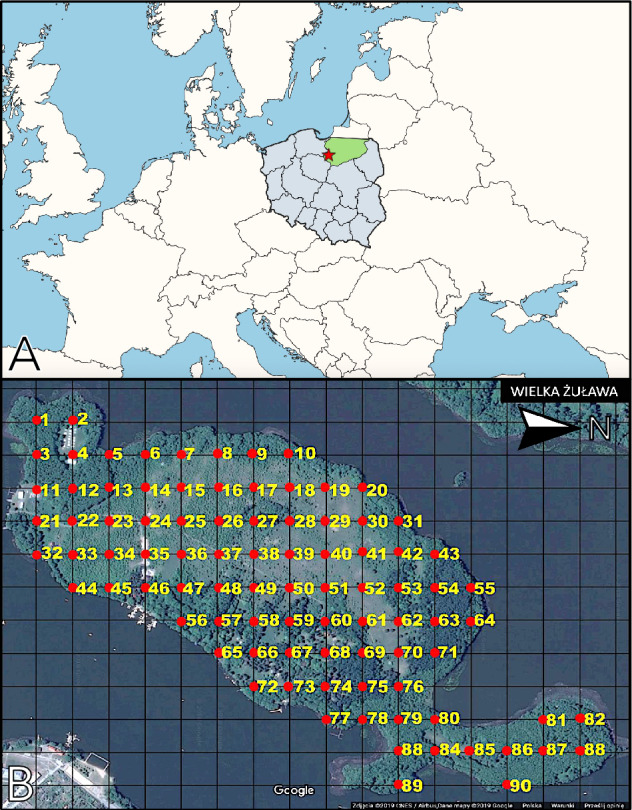
The study site and sampling grid. (**A**) The location of the Wielka Żuława island (star) in the Warmia-Masuria region (green) in Poland. Map created in QGIS (v3.28.1-Firenze; https://www.qgis.org/). (**B**) A satellite image of the Wielka Żuława island overlayed with the sampling grid applied in the case study. Consecutive samples (red dots) in the grid were collected at 100 m distance. Satellite image: ©2019 CNES/Airbus; ©2019 Google (https://maps.google.com).

To prepare example local-scale distribution maps of soil fungi, a case study was carried out on Wielka Żuława island on the Jeziorak lake in the Warmia-Masuria region in Poland (Fig. 1A). From across the island, 90 samples were collected in a regular square grid^28^ at 100-m intervals (Fig. 1B). Each sample comprised of soil mixed from three sampling points 1 m apart from each other, forming an equilateral triangle with its center on the grid node. A 2 × 10 cm cylindrical soil core was collected at each of these sampling points. To prevent sample cross-contamination, the sampling tools were sterilized using ethyl alcohol and a blow torch following the collection of each sample. Samples were placed in separate zip-lock plastic bags and stored in -20 centigrade until further processing. During sample collection, the surrounding vegetation type and tree species were recorded for each sample (Appendix 1). All sampling took place in June 2021.

### Soil chemical analysis

Soil chemical analysis was performed independently for each of the 90 samples. Soil pH was measured in soil–water suspension using a Mettler Toledo FE20 benchtop pH meter. Kjeldahl N was measured using a FOSS TECATOR 2300 Kjeltec Analyzer Unit (application note AN 300). Total C and organic C were measured using a LECO RC612 Multiphase Determinator. Dionex ICS-1100 Ion Chromatography System was used to analyze N–NO_3_, N–NH_4_, and P (Olsen). For N–NO_3_, Dionex Seven Anion Standard II was used to calibrate, and the Dionex IonPac™ AS9-HC column was used for analysis. For N–NH_4_, Dionex Six Cation Standard II was used to calibrate, and the Dionex IonPac™ CS12A column was used for analysis. For P (Olsen), the Dionex IonPac™ AS9-HC column was used for analysis. Cation content (K, Mg, Ca) was measured using the Varian AA280FS spectrometer (Fast Sequential Atomic Absorption Spectrometer 280; Varian, Australia). The analyses were verified using certified reference materials: ISE sample 995 and ISE sample 859. Soil chemical analyses were conducted at the Laboratory of Ecochemistry and Environmental Engineering, Institute of Botany, Polish Academy of Sciences.

### Sample analysis

The DNeasy PowerSoil (Qiagen) kit was used to extract and purify total soil DNA from each sample. The primers gITS7 (5′-GTG ART CAT CGA RTC TTT G-3′) and ITS4 (5′-TCC TCC GCT TAT TGA TAT GC-3′) were used for the PCR reactions to amplify the standard fungal barcoding gene ITS2^29,30^. Three independent PCR amplifications were conducted for each sample. The products of the three PCRs were then brought together to form a library from each sample for the following analyses. The 90 libraries were sequenced in paired-end mode on the high-throughput Illumina MiSeq platform (minimal run length: 2 × 250 base pairs). The sequencing was conducted by Novogen (novogen-layers.com). The Illumina sequencing results were deposited in the Sequence Read Archive (https://www.ncbi.nlm.nih.gov/sra), accession numbers: SRR24200866–SRR24200782 under the BioProject PRJNA956702.

### Data analysis

The sequencing results were filtered to remove low-quality and chimeric sequences. The cleaned sequences were grouped to form operational taxonomic units (OTUs) based on a ≥ 97% identity threshold. FLASH (v1.2.7; http://ccb.jhu.edu/software/FLASH/), UCHIME (http://www.drive5.com/usearch/manual/uchime_algo.html), QIIME (v1.7.0; http://qiime.org/scripts/split_libraries_fastq.html)^31^ and Uparse (Uparse v7.0.1090; http://drive5.com/uparse/) platforms were used to perform these operations^32–34^.

The taxonomic position of the resulting OTUs was established by comparing each OTU representative sequence with reference sequences using the blastall (v2.2.25) algorithm and the UNITe (v8.2) database (https://unite.ut.ee/)^35^. Phylogenetic relationships between the OTU representative sequences were established after aligning the sequences using the MUSCLE algorithm (v3.8.31; http://www.drive5.com/muscle/)^36^. The OTUs count was normalized based on a standard corresponding to the sample containing the lowest number of sequences. Finally, each OTU was assigned to a trophic mode and guild using the FunGuild platform^37^. Correlations between soil chemical variables, between the abundance of individual fungal guilds, and between fungal guilds and soil properties were calculated based on Spearman’s rank correlation coefficient in R (v4.2.2) using the ‘corrplot’ package^38^.

### Map preparation

The local-scale distribution of individual fungal groups was represented as 2-D histograms and georeferenced raster grids. The 2-D histograms were created in Excel by assigning the measured sequence counts for individual groups of fungi to the respective sampling points, with the X and Y axes representing the relative location (Appendix 1). Spatial autocorrelation in the data sets was evaluated by calculating the global Moran’s I^39^. The neighboring cells were assigned the weight (*w*) of 1, and remote cells the weight of 0 based on the rook’s definition of contiguity (the same weight matrix was used for all tested fungal groups); *n* represents the number of all cells and *x* represents the measured values. The significance of the calculated Moran’s I was assessed using the Monte Carlo permutation test (1000 permutations).

$$I = \frac{{n\mathop \sum \nolimits_{i = 1}^{n} \mathop \sum \nolimits_{j = 1}^{n} w_{ij} \left( {x_{i} - \overline{x}} \right)\left( {x_{j} - \overline{x}} \right)}}{{\left( {\mathop \sum \nolimits_{i = 1}^{n} \mathop \sum \nolimits_{j = 1}^{n} w_{ij} } \right)\left( {\mathop \sum \nolimits_{i = 1}^{n} \left( {x_{i} - \overline{x}} \right)^{2} } \right)}}$$

In QGIS (v3.28.1-Firenze; https://www.qgis.org/) software^40^, a shapefile layer was created, with all sampling locations added as 90 individual points. The sequence counts for individual groups of fungi at each sampling location were assigned to the respective points as their point attributes. The shapefile layer was used as a basis for spatial interpolations. Three methods of raster grid interpolation were tested in preparing the distribution maps: IDW, multilevel B-spline, and Kriging interpolation (ordinary Kriging; the variogram function was fitted for each set of data independently based on the indicated determination value). The cell size was set for all interpolation algorithms to 0.0001°, resulting in 190 columns and 162 rows. All interpolations were performed using SAGA (v8.4.1) GIS software^41^. All the resulting georeferenced raster grids were imported to QGIS software for further management, editing, and analysis.

The effects of reduced sampling effort (RSE) on the resulting fungi distribution maps were tested by interpolating maps based on a limited number of sample data points. In all these simulations, the remaining points formed regular grids. Three levels of RSE were simulated: low (using half of the samples; 1/2 N), medium (using a quarter of the samples; 1/4 N), and high (using 1/9 of the samples). Two sets of maps were created for both low- and medium-level RSE simulations, each set using a different selection of data points.

### Map validation

To evaluate the number, size, and shape of similar fungi abundance patches in the interpolated maps, tested raster grids were segmented using k-means clustering. Using QGIS, the tested raster grids were set to the ‘single-band grey’ setting, representing all values on a monochromatic grey scale. K-means clustering of the prepared maps was performed in MATLAB (v9.13.0, R2022b; https://www.mathworks.com) software using the ‘imsegkmeans’ function (https://www.mathworks.com/help/images/ref/imsegkmeans.html)^42^. All tested maps were segmented for k = 2 (dividing them into two categories of regions) and k = 3 (three categories of regions). The performance of interpolations was evaluated by calculating their root mean square error (RMSE). Each tested interpolation was repeated using 80% of randomly selected data points, and the *n* measured (*x*) and interpolated values ($\hat{x}$) at the remaining points were collected using the Point Sampling Tool QGIS plugin (v0.5.4)^43^ compared using the following equation:

$$RMSE = \sqrt {\frac{{\mathop \sum \nolimits_{i = 1}^{n} \left( {\hat{x}_{i} - x_{i} } \right)^{2} }}{n}}$$

To evaluate the accuracy of RSE simulations as compared to the maps prepared based on all available data points, difference raster grids were prepared. Difference raster grids were calculated by subtracting individual simulated RSE raster grids from the analogous raster grid interpolated from all available points. For each resulting difference raster grid, the average value, standard deviation (SD), and the sum of squares were calculated. The arithmetic operations on raster grids were performed in QGIS software.

The distribution patterns of selected fungal groups were compared with the basic features of the Wielka Żuława landscape. The raster grids were set to 40% opacity and superimposed on a general-use map of the island (OpenStreetMaps; https://www.openstreetmap.org).

## Results

Soil analyses revealed high differences in chemical properties among the sampling points (Table 1). The average pH was strongly acidic (5.28), with 68.9% of samples being strongly acidic (pH < 5.5). Most of the analyzed soil chemical variables were positively correlated (Appendix 2). The exception to this was phosphorus, which negatively correlated with nitrogen (Kjeldahl and NH_4_) and carbon (total and organic). The main types of vegetation on the island were mature trees (predominately ECM species) and grasses (Appendix 1).

**Table 1:** Lowest, average, and highest values of measured soil chemical variables recorded from the study samples.

	N (Kjeldahl) (%)	C_tot._ (%)	C_org._ (%)	N-NO_3_ (ppm)	N-NH_4_ (ppm)	K (ppm)	Mg (ppm)	Ca (ppm)	P (Olsen) (ppm)	pH
Lowest value	0.052	0.520	0.492	0.019	0.544	21.03	16.35	82.42	0.479	4.12
Average	0.290	4.991	4.816	8.327	4.979	101.57	148.60	1511.70	13.787	5.28
Highest value	1.939	30.877	30.727	213.266	74.356	394.05	1256.15	13,058.30	51.789	7.22

**Figure 2:**
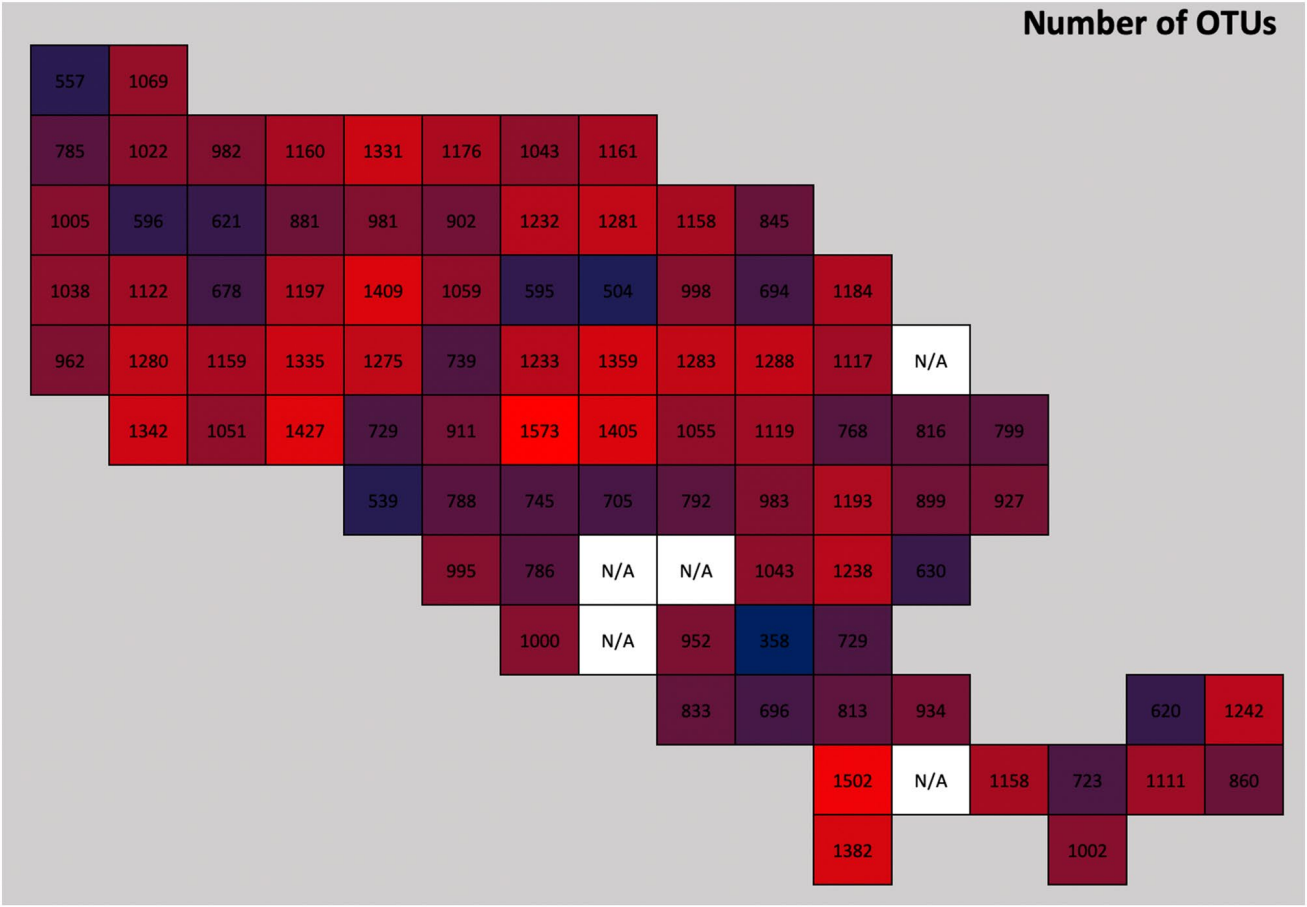
A 2-D histogram illustrating the number of distinct fungal OTUs detected from each soil sample.

Sequencing was successful for 85 out of the 90 samples. No data were obtained for samples 43, 67, 68, 73, and 84. Most distinct fungal OTUs (1573) were identified from sample 49, while sample 75 contained the lowest number of OTUs (358) (Fig. 2). Overall, the fungi identified on the Wielka Żuława island comprised 10,387 OTUs, 965 genera, 373 families, 158 orders, and 12 phyla. The fungi were classified into 32 trophic guilds. While both the most abundant genus (*Penicillium*) and the most abundant family (Aspergillaceae) are both predominately saprotrophic, overall, the most abundant trophic guild recorded were ectomycorrhizal (ECM) fungi. The most abundant genus of ECM fungi was *Russula* (Table 2).

**Table 2:** Most abundant groups of soil fungi on the genus, family, and trophic guild levels recorded in the case study.

Rank	Name	Moran’s I	*p*
Genus			
1.	*Penicillium*	0.080	N.S
2.	*Russula*	0.025	N.S
3.	*Inocybe*	0.027	N.S
4.	*Oidiodendron*	0.000	N.S
5.	*Exophiala*	**0.156**	** < 0.05**
6.	*Tomentella*	–0.007	N.S
7.	*Saitozyma*	0.090	N.S
8.	*Mortierella*	**0.195**	** < 0.01**
9.	*Cortinarius*	0.058	N.S
10.	*Trechispora*	-0.044	N.S
Family			
1.	Aspergillaceae	0.051	N.S
2.	Russulaceae	0.015	N.S
3.	Myxotrichaceae	0.000	N.S
4.	Inocybaceae	0.027	N.S
5.	Herpotrichiellaceae	0.123	N.S
6.	Pyronemataceae	**0.238**	** < 0.01**
7.	Thelephoraceae	–0.002	N.S
8.	Trimorphomycetaceae	0.090	N.S
9.	Nectriaceae	**0.156**	** < 0.05**
10.	Hymenogastraceae	–0.051	N.S
Trophic Guild			
1.	Ectomycorrhizal (ECM)	**0.143**	** < 0.05**
2.	Undefined saprotroph	0.021	N.S
3.	Wood saprotroph	–0.006	N.S
4.	Dung saprotroph	0.006	N.S
5.	Plant pathogen	0.102	N.S
6.	Endophyte	**0.237**	** < 0.001**
7.	Fungal parasite	**0.129**	** < 0.05**
8.	Animal pathogen	**0.150**	** < 0.05**
9.	Soil saprotroph	0.083	N.S
10.	Ericoid mycorrhizal	0.009	N.S

For each group, the spatial autocorrelation coefficient Morran’s I and its significance *p* is provided (*N.S.* not significant).

**Figure 3:**
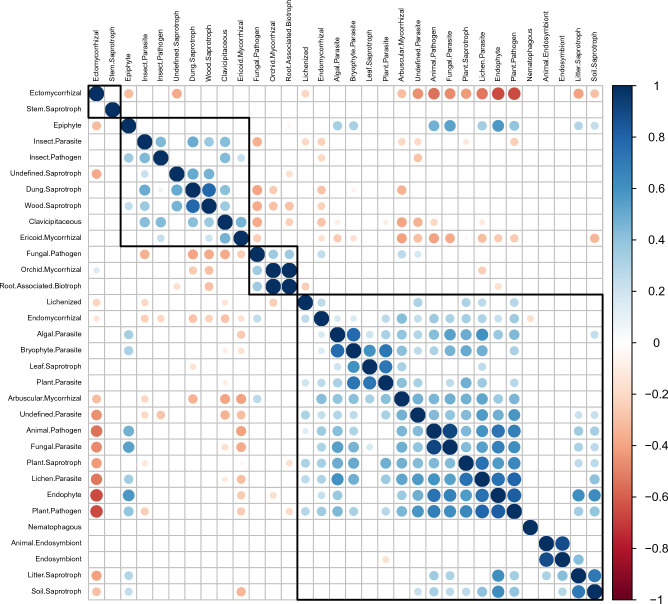
Hierarchically clustered Spearman’s rank correlation matrix of the soil fungi trophic groups identified on the Wielka Żuława island. Only significant correlations are presented.

Most fungal trophic guilds were discovered to form clusters based on the positive correlations in their distribution (Fig. 3). These clusters, however, did not contain ECM fungi, the most dominant guild. The distribution of ECM fungi correlated negatively with the distribution of several of the other trophic guilds, particularly the guilds forming the largest cluster. Stem saprotrophs and nematophagous fungi showed no significant correlations to the other guilds in their distribution. Only half of the fungal trophic guilds’ distribution was significantly correlated with the measured soil chemical variables. Notably, ECM fungi distribution was not found to corelate with any soil parameters. Of soil chemical variables, pH and Ca were found to be significantly correlated with the distribution of the highest number of fungal trophic guilds (7 guilds each; Appendix 2).

Positive spatial autocorrelation was found in four out of the ten most abundant trophic guilds (Table 2). The highest spatial autocorrelation characterized the distribution of fungal endophytes (I = 0.237, *p* < 0.001). Two out of ten most abundant families (Pyronemataceae and Nectriaceae) and two out of ten most abundant genera (*Exophiala* and *Mortierella*) displayed significant spatial autocorrelation on the sampled scale.

The B-spline interpolation consistently produced the highest RMSE values. The IDW method produced the lowest RMSE values for the fungal groups that did not display significant spatial autocorrelation on the sampled scale. The Kriging interpolation produced the lowest RMSE values for the fungal groups with spatially autocorrelated distribution (Table 3). However, the Kriging interpolation is the only of the tested methods to return altered values for the sampling points used for interpolation.

**Table 3:** Root mean square error (RMSE) of the tested interpolation methods for the ten most abundant trophic guilds of soil fungi recorded in the case study.

Rank	Name	IDW	BS	K	Significant spatial autocorrelation
1.	Ectomycorrhizal (ECM)	0.239	0.282	0.237	Yes
2.	Undefined saprotroph	0.137	0.157	0.140	No
3.	Wood saprotroph	0.150	0.172	0.171	No
4.	Dung saprotroph	0.154	0.167	0.159	No
5.	Plant pathogen	0.076	0.098	0.080	No
6.	Endophyte	0.056	0.072	0.055	Yes
7.	Fungal parasite	0.054	0.075	0.053	Yes
8.	Animal pathogen	0.054	0.073	0.052	Yes
9.	Soil saprotroph	0.110	0.113	0.112	No
10.	Ericoid mycorrhizal	0.034	0.044	0.035	No

**Figure 4:**
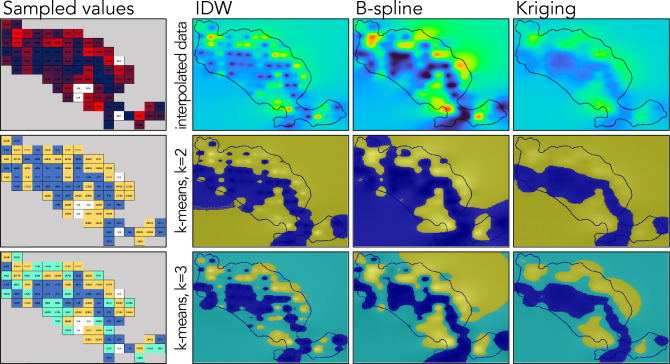
The number of ectomycorrhizal (ECM) fungi reads in the collected samples (sampled values) on the Wielka Żuława island and ECM fungi distribution maps prepared with inverse distance weighted (IDW), B-spline, and Kriging interpolation methods. Stratified maps prepared with k-means clustering for k = 2 (relative high- and low-abundance) and k = 3 (relative high-, medium-, and low-abundance) illustrate the relative high (yellow), medium (cyan), and low (blue) abundance regions delineated with each interpolation method.

**Figure 5:**
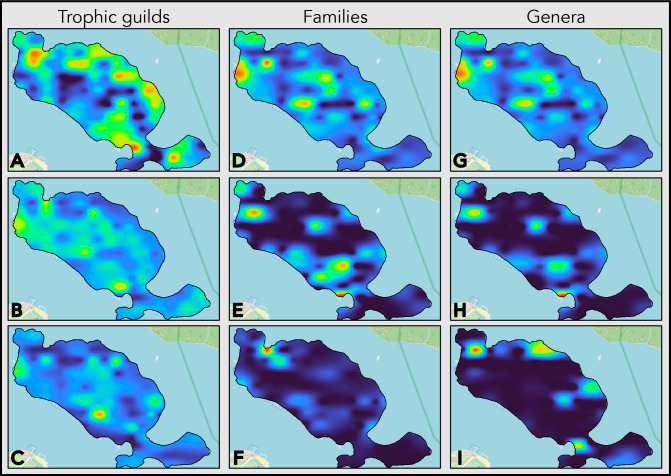
B-spline interpolated soil fungi distribution maps of most abundant fungal groups on the Wielka Żuława island on the trophic guild, family, and genus levels. (**A**) ectomycorrhizal; (**B**) undefined saprotrophs; (**C**) wood saprotrophs; (**D**) Aspergillaceae; (**E**) Russulaceae; (**F**) Myxotrichaceae; (**G**) *Penicillium*; (**H**) *Russula*; (**I**) *Inocybe*.

The method most accurate at delineating discrete regions of similar relative abundance of fungi was B-spline (Fig. 4). Using the IDW algorithm resulted in maps with no continuity between the data points in regions of similar abundance. This limited continuity is more apparent in maps stratified into discrete regions using k-means clustering. Kriging-based maps portray major features of fungal distribution (i.e., large high- vs. low-abundance regions) but omit smaller patches. Using B-spline allows for the successful preparation of distribution maps for any discrete group of soil fungi on any level of organization (Fig. 5).

**Figure 6:**
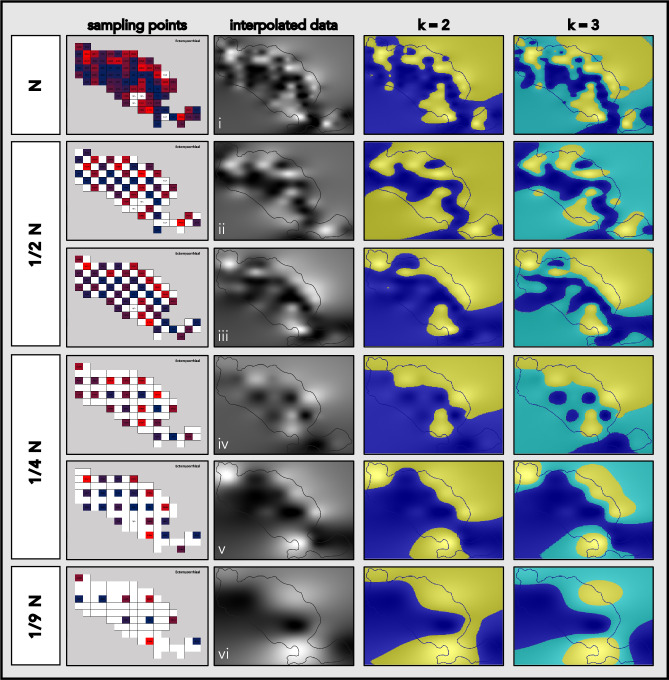
Reduced sampling effort (RSE) simulations of the ectomycorrhizal (ECM) fungi distribution mapping. The 2-D histograms indicate the samples used for subsequent interpolations. B-spline interpolated distribution maps were stratified with k-means clustering for k = 2 (relative high- and low-abundance) and k = 3 (relative high-, medium-, and low-abundance) to illustrate the relative high (yellow), medium (cyan), and low (blue) abundance regions indicated in each RSE simulation. (**i**) map interpolated with all available data (N; distance between consecutive data points = 100 m); (**ii** and **iii**) maps interpolated with 1/2 of the available data (1/2 N; distance between consecutive data points = 141 m); (**iv** and **v**) maps interpolated with 1/4 of the available data (1/4 N; distance between consecutive data points = 200 m); (**vi**) map interpolated with 1/9 of the available data (1/9 N; distance between consecutive data points = 300 m).

**Figure 7:**
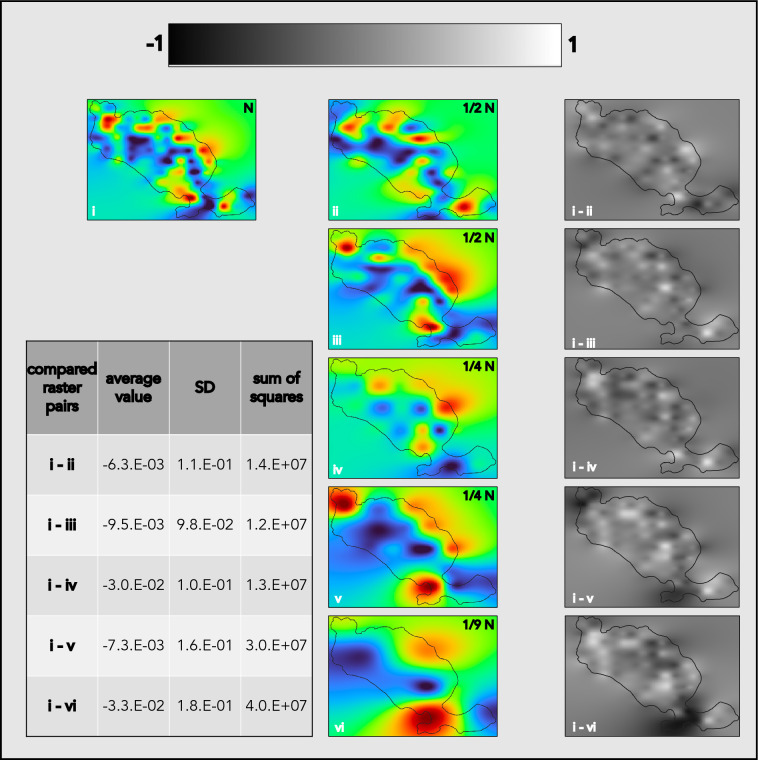
Difference raster grids for the reduced sampling effort (RSE) simulations of the ectomycorrhizal (ECM) fungi distribution mapping. At each point of the raster grid, the values of RSE raster grids (ii–vi) were subtracted from the raster grid interpolated with all available data (i). In the difference raster grids, darker colors indicate underestimation and lighter colors overestimation of abundance in comparison to raster i. For each difference raster grid, its average value, standard deviation (SD), and sum of squares are listed.

The resolution of detected fungal distribution features was affected by RSE (Fig. 6). For all tested RSE simulations, the two major regions of high ECM fungi abundance (on the island’s west coast and at the base of the peninsula in the north) were detected. Distribution patches smaller than the distance between the sampling points were either missed or exaggerated (e.g., the region of high ECM fungi abundance on the northern peninsula) depending on the sampling grid positioning. With a larger distance between sampling points, the potential role of sampling grid positioning increases. This is illustrated by comparing distribution maps interpolated from all collected samples with the RSE maps (Fig. 7). For ECM fungi, sums of squares for difference raster grids were comparable for maps prepared based on low (1/2 N) RSE (i–ii: 1.4e+7 and i–iii: 1.2e+7), but differed by a factor of two for maps prepared based on medium (1/4 N) RSE (i–iv: 1.3e+7 and i–v: 3.0e + 7).

**Figure 8:**
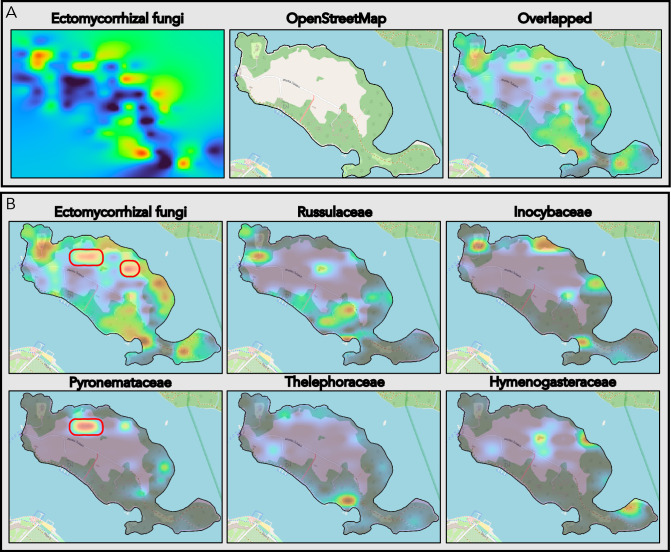
The distribution of ectomycorrhizal (ECM) fungi on the Wielka Żuława island juxtaposed with the landscape of the island and the distribution of fungal families including ECM taxa. (**A**) An overlay of the ECM fungi distribution map and OpenStreetMap of Wielka Żuława. Most of the regions of high ECM fungi distribution overlap with forested areas (dark green on the OpenStreetMap layer). (**B**) Distribution maps of ECM fungi and the 5 most abundant fungal families to include ECM taxa: Russulaceae, Inocybaceae, Pyronemataceae, Thelephoraceae, and Hymenogasteraceae. Red contours indicate regions of high ECM fungi distribution outside of the forested areas. Comparing the distribution maps of all ECM fungi and of individual families reveals that Pyronemataceae are the main contributor to the indicated regions.

Overlaying the fungi distribution maps and the OpenStreetMap of the Wielka Żuława island reveals relationships between the landscape features and the abundance of mapped fungi. Most of the high-abundance patches of ECM fungi overlapped with the forested parts of the island, indicated on the OpenStreetMap by dark green (Fig. 8A). Two patches of high ECM fungi abundance were placed outside the forested region as indicated by the OpenStreetMap. Analyzing the distribution of the five most abundant fungal families comprising ECM fungi revealed Pyronemataceae as the main contributor to one of these patches (Fig. 8B), with the other families largely found in the forest regions.

## Discussion

### Environmental conditions and soil fungi at the study site

The environmental conditions observed at the Wielka Żuława island are typical for this part of Europe. The average soil pH in Europe is acidic, except for the Mediterranean region, where most soils are alkalic^44^. In Poland, most soils have a pH below 5.5^45^. Examining the spatial distribution of the measured soil chemical variables suggests some degree of differentiation between the island coast and the island center (Appendix 1). The recorded tree species composition on the island is consistent with the forests characteristic of the Warmia-Masuria region^46^. Comparing the soil chemical variables and the recorded vegetation at the sampling points reveals some tentative relationships. Regions of high phosphorus concentration conspicuously overlap with regions dominated by grasses, with few or no trees. Regions of low calcium concentration largely coincided with the distribution of *Pinus sylvestris*, the only conifer species on the island.

The observed environmental variables provided limited insight into the soil fungal community on the Wielka Żuława island. Half of the fungal trophic guilds (including the most abundant guild, ECM fungi) did not significantly correlate with the soil chemical variables. Moreover, the significant correlations were characterized by relatively low correlation coefficients. The two soil chemical variables significantly correlated with the largest number of fungal trophic guilds, pH and Ca, are known to be important predictors of fungal diversity. A study by Tedersoo et al.^47^ indicated soil pH as the primary driver of soil fungal diversity in the Baltic region of Europe. The same study found calcium concentration to be the second most important soil chemical variable affecting fungal distribution, outweighing such variables as P, K, Mg, C, and N concentrations. Although the role of soil chemistry in shaping the local soil fungi communities is significant, the low correlation coefficients between the soil chemical variables and the abundance of individual fungal groups suggests that other factors shaping the community may also be at play. Identifying and understanding these factors can (at least in part) be achieved by mapping the soil fungi and analyzing their distribution.

### Use of interpolation in landscape-scale fungi distribution mapping

Using interpolation to map spatial variables is a well-established approach in fields like geology^24,26,48^ or meteorology^27,49^, also applied in some areas of ecology^25,50,51^. In continuous variables, the values change gradually, and thus the neighboring values are not independent of one another. This spatial autocorrelation^26^ enables predicting the values of a studied variable based on the observed surrounding values. The influence of a variable’s value at one point on its values at other points usually decreases with distance and, as such, can only be observed on certain scales^51^. The level of soil fungal biomass is known to be a continuous variable spatially autocorrelated on the landscape scale^50^. Studies of ECM fungi based on ECM root tips indicated spatial autocorrelation of the species community composition on spatial scales varying from 2.6 to 25 m, depending on the study site and the fungal species^52^. However, the free mycelium and rhizomorphs^53,54^ may grow outward from the root tips extending for meters^55,56^. Consequently, the scale at which the ECM fungi in soil display spatial autocorrelation should noticeably exceed the scale at which spatial autocorrelation is observed for the ECM root tips. Other groups, such as saprotrophic and pathogenic fungi, may form continuous hyphal patches much larger than these of ECM fungi^56^.

At the spatial scale sampled in the case study, significant spatial autocorrelation was observed only for the minority of tested groups of soil fungi. This could be caused either by a large error in the sampled values or the spatial autocorrelation relevant only on a scale smaller than the minimum distance between the collected samples^25,57^. The latter seems to be the case here. The strong correlation between soil fungi relative abundance and the read counts of respective barcodes in Illumina metasequencing^58^ suggests the reliability of the values observed for the sampled points. However, the minimum separation distance between samples in the case study was 100 m, exceeding the scale of observable autocorrelation for individual ECM fungal species mentioned above. Accordingly, on the genus level, significant spatial autocorrelation was observed only for predominately pathogenic *Exophiala*^59^ and predominately saprotrophic *Mortierella*^60^. While Pyronemataceae, one of the families for which significant spatial autocorrelation was observed on the sampled scale, does include ECM taxa, most of its species are saprotrophic^61^. Most groups of fungi spatially autocorrelated on the sampled scale were on the trophic guilds’ level, interestingly including ECM fungi. This could be explained by the smaller, discrete patches of individual fungal taxa belonging to the same guild displaying a level of functional redundancy^62^ and responding to the environmental conditions in similar ways. As a result, they form larger, ‘effective’ patches that display significant spatial autocorrelation on the sampled scale. It is important to note that while observing and characterizing the spatial autocorrelation is necessary for Kriging interpolation, IDW and B-spline methods may be used just with the assumption of the interpolated variable being spatially autocorrelated^57^.

Each of the compared interpolation methods presented a distinct set of advantages and disadvantages. Based on the RMSE scores, IDW and Kriging were the best predictors of soil fungi relative abundance values. While Kriging interpolation performed better for groups of fungi displaying spatial autocorrelation on the sampled scale, IDW performance for these fungi was similar, differing by only 1–3%. For the groups of fungi where spatial autocorrelation on the sampled scale was not observed, IDW performed much better than Kriging interpolation. This observation is consistent with Schloeder et al.^24^, who suggested that in the case of sparsely distributed data on soil properties, IDW and Kriging performance are comparable. B-spline, followed by Kriging (for the groups of fungi in which spatial autocorrelation was observed at the sampled scale), outperformed IDW in terms of depicting patches and regions of the fungi distribution, especially for stratified maps. This is also consistent with the characteristics of IDW interpolation, which makes predictions only based on the variable value and distance from a known data point, not considering the values of the surrounding points^26,48,57^. Overall, each interpolation method seems to be best suited for different applications. IDW is most suitable for predicting the fungi relative abundance at unsampled points if the studied soil fungi communities do not display spatial autocorrelation on the sampled scale or if it is uncertain whether they do. B-spline is most suitable for predicting and visualizing the spatial patterns of fungal distribution. Kriging interpolation outperforms IDW in predicting the fungi relative abundance values and predicting the spatial patterns of fungal distribution if the spatial autocorrelation can be characterized on the sampling scale.

### Uses for landscape-scale mapping of soil fungi

In conventional studies of soil fungal ecology at the landscape scale, only a limited number of samples are collected. Similarly, only selected environmental variables are being measured. Both the distribution of samples and the selection of measured environmental variables tend to be informed by the study objectives and hypotheses. Studies focusing on the effects of specific environmental variables may collect the samples alongside these studied variables’ gradients^63–66^. If the research objectives are investigating the ecological relationships between soil fungi and other organisms, e.g., selected plants, the samples may be collected from the vicinity of these plants^19,22^. In cases where general characterization of the soil fungal communities in a studied landscape is needed, sampling often is unrelated to the landscape features and effectively pseudo-random^67–69^. This conventional, ‘purposeful’ approach to sampling design is effective in answering the original questions posed in the respective studies. However, it can limit the potential reusability of the collected data.

Mapping the distribution of soil fungi in a landscape facilitates post-hoc testing for correlations between the fungal distribution and environmental variables. Studies implementing conventional sampling designs may lack data on the fungal abundance across environmental variable gradients that were not considered when the study was designed. Even if a wide range of values is measured and reflected in the gathered samples for a variable that the study did not initially attempt to focus on, that variable may be correlated with one for which the study was planned (e.g., soil water content and soil organic matter similarly distributed in the samples of Aučina et al.^65^ or soil pH overlapping with the tree community composition in the study of Wilgan et al.^19^). Separating the effects of multiple environmental variables correlated with each other could be strenuous if possible. If the relative abundance of soil fungal groups is known for each specific point of a landscape, that abundance can be juxtaposed with independently tested environmental variables. Moreover, if the fungi distribution map is superimposed on other maps illustrating the local landscape features, potential factors affecting the fungal distribution which were not previously considered could be noticed. All this may be done either by the researchers who first prepared the distribution map or by any different group.

Compared to studies implementing the conventional sampling design, soil fungi distribution maps can be more easily used by other researchers. In some cases, analyzing the effects of landscape features and environmental variables omitted by the team that prepared the map would not even require revisiting the study site. The mapped data of fungal abundance could be compared with other maps and satellite images^70^ of the study site to identify previously overlooked factors shaping the local distribution of soil fungi. The ability to perform additional studies on a given site without subsequent visits may be relevant when visiting the site is dangerous^71^ or disturbs the local environment^72,73^. Additionally, producing landscape-scale maps of soil fungi distribution makes it easier to make landscape-scale environmental studies of soil fungi replicable and verifiable^20,21^. Even if the precise location of sampling points in the study which is being verified is not available, a repeated mapping of the same landscape should reveal similar features in the distribution of individual fungal groups.

Landscape-scale maps of soil fungal distribution have numerous potential practical applications. They could be used in designing effective nature conservation areas. Soil fungi are essential for the functioning of local habitats^3,5^, and knowing their distribution may be useful in determining the size and shape of the conservation area. Moreover, some rare fungal taxa are potentially worth preserving themselves^74^, and their distribution should be considered. Maps of soil fungi distribution could also be used to better understand and manage urban green spaces^75^. The importance of urban green spaces in cities around the world is well recognized^76^. The local fungal communities, which include pathogenic and symbiotic fungi, play an essential role in the condition of these green spaces^77,78^. Other areas where maps of landscape-scale fungi distribution may be helpful are planning and evaluating the impact of local infrastructure projects, agriculture, or forestry.

### Potential issues and solutions

Probably the biggest issue limiting the applicability of the proposed methodology is its cost. Compared to conventional sampling designs, the number of samples required for landscape-scale mapping of the soil fungi distribution is notably higher. While the conventional approach will remain a cheaper alternative, the per-sample costs in molecular studies are continually decreasing^30^. As a result, the proposed mapping method will become increasingly available to diverse research teams around the world. Moreover, the costs of soil fungi distribution mapping can be optimized by adjusting the sampling intensity. The presented case study on Wielka Żuława indicates that the distance between collected samples in the sampling grid affects the map resolution and the size of detectable fungi distribution patches. Accordingly, adjusting that distance while preparing individual maps should happen considering the map's purpose. This could range from increasing the sampling intensity (in applications concerned with the fine-scale details of soil fungi distribution or the precise distribution of fungal groups displaying spatial autocorrelation only on small spatial scales) to decreasing it (in applications where only the general features of the fungi distribution, i.e., the spatial arrangement of stratified regions of individual fungal groups, are needed). A similar approach of reducing the sampling intensity depending on the intended use for the interpolated maps was also suggested in other fields, e.g., mapping lake sediments^48^.

Molecular studies of fungal communities and their diversity are susceptible to several forms of bias. First, the effectiveness of DNA isolation and amplification varies between individual fungal taxa^30,79–81^. As a result, some recalcitrant taxa may be relatively underrepresented in the libraries prepared for downstream sequencing applications. One solution that could partially addresses this issue is to use single-molecule real-time sequencing platforms, avoiding the PCR amplification stage. However, employing these platforms may be expensive; compared to Illumina sequencing used in the present study, PacBio could cost up to ten times more per base pair^30^. Next, distinct fungal taxa are known to contain different numbers of barcode gene repetitions in their genomes^30,58,82^. Using the proportion of barcode gene reads in a sample as a direct proxy for the fungal relative abundance can, in some cases, lead to over- or underestimations of the community share of certain fungi. This issue can be addressed if the estimated relative abundance of fungi is corrected for the number of barcode gene repetitions in individual taxa. Studies indicate that such a corrected number of reads is an accurate predictor of fungal relative abundance^82,83^. Finally, it is important to remember that the proposed method produces maps of relative rather than absolute abundance. To convert to absolute abundance, additional measures of total fungal biomass (e.g., ergosterol concentration^50,84^) should also be collected. Relative abundance maps are most accurate for individual groups of fungi and visualize patches of their (relative) low and high abundance. Comparing the mapped relative abundance between distinct groups of fungi may lead to important insights, but the aforementioned biases should be recognized during these insights’ interpretation.

In the present study, three widely used interpolation algorithms were compared for fungi distribution map preparation. Although the B-spline interpolation method produces practical results, the authors see future potential in replacing any classical interpolation methods with machine learning solutions^85^. A neural network trained specifically with data on landscape-level fungi distribution could potentially produce more accurate results compared to a general-use interpolation algorithm. Collecting suitable training data and developing a neural network for this purpose would be an interesting future development.

## Supplementary Information

Supplementary Information 1.

Supplementary Information 2.

## Data Availability

The datasets generated and analyzed during the current study are available in the Sequence Read Archive (SRA) repository. (BioProject PRJNA956702 https://www.ncbi.nlm.nih.gov/sra/PRJNA956702. Acession numbers: SRR24200866–SRR24200782).
