# Supplementary material for: Landscape-scale mapping of soil fungal distribution: proposing a new NGS-based approach
Source: Sci Rep. 2023 Jun 24;13:10280. doi: 10.1038/s41598-023-37538-7 (PMC10290699; doi:10.1038/s41598-023-37538-7)
Supplement: Supplementary file 2 — Supplementary Information 2. [file 41598_2023_37538_MOESM2_ESM.pdf]

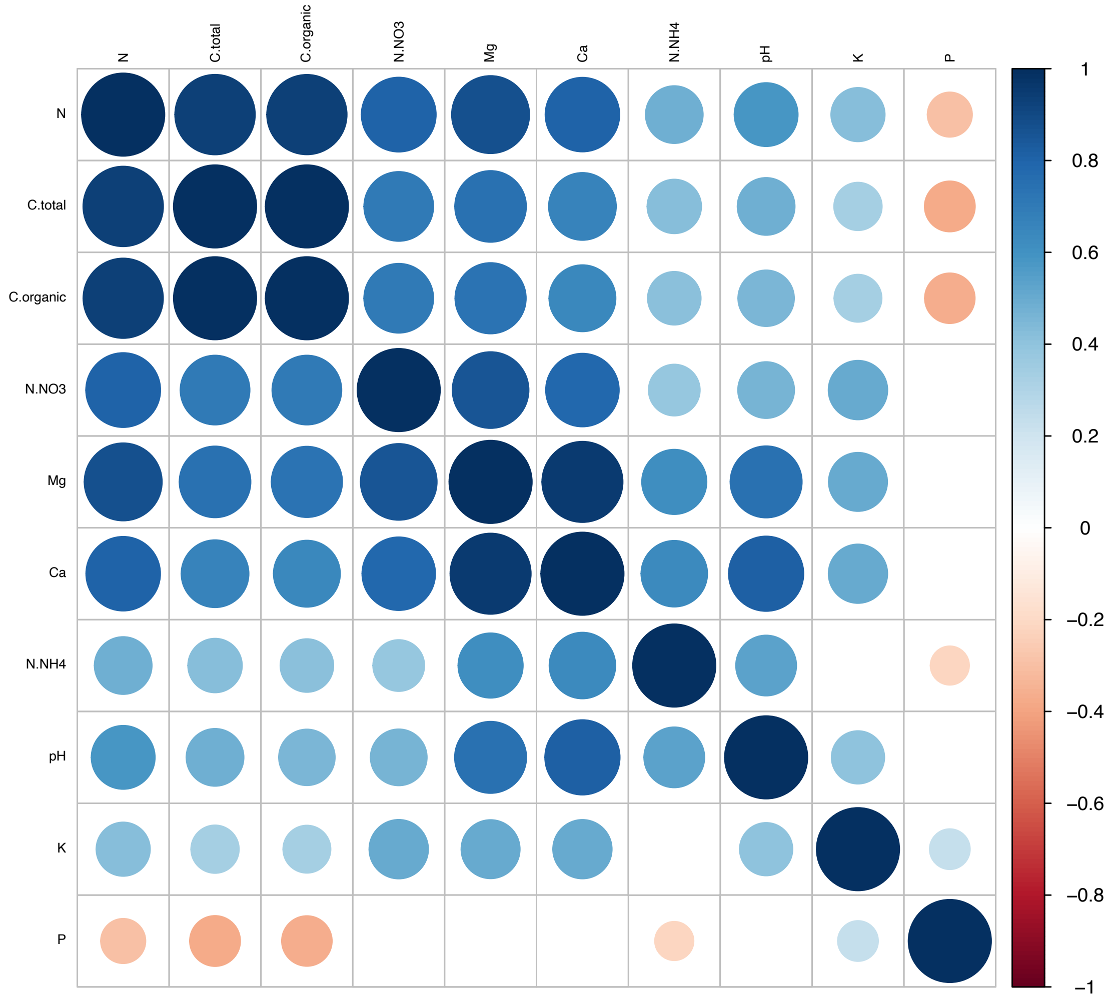

Appendix 2A: Spearman's rank correlation matrix of the soil chemistry variables measured in the samples from the Wielka Żuława island. Only significant correlations are presented.

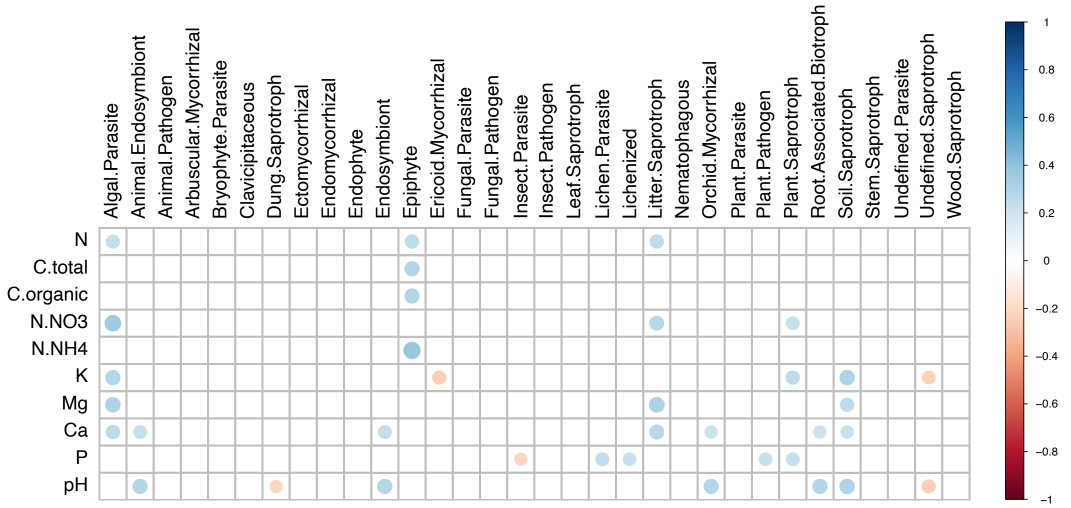

Appendix 2B: Spearman's rank correlation matrix of the soil chemistry variables vs. fungal trophic guilds identified on the Wielka Żuława island. Only significant correlations are presented.
